# Supplementary figures and images for: Effects of T cell leptin signaling on systemic glucose tolerance and T cell responses in obesity
Source: PLoS One. 2023 Jun 5;18(6):e0286470. doi: 10.1371/journal.pone.0286470 (PMC10241364; doi:10.1371/journal.pone.0286470)

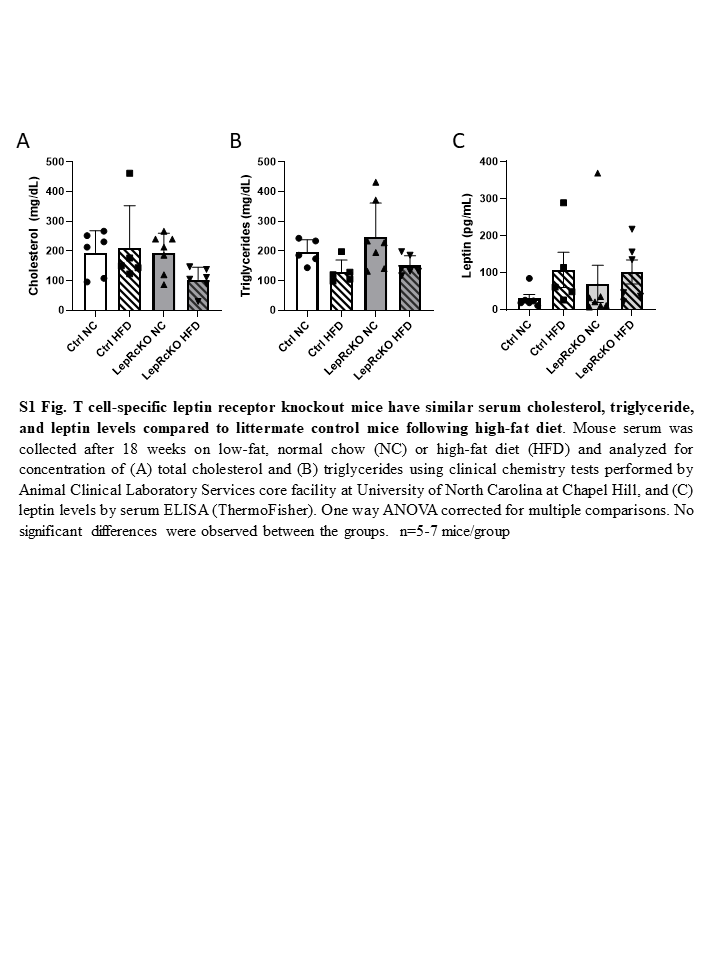

Supplement: S1 Fig — Mouse serum was collected after 18 weeks on low-fat, normal chow (NC) or high-fat diet (HFD) and analyzed for concentration of (A) total cholesterol and (B) triglycerides using clinical chemistry tests performed by Animal Clinical Laboratory Services core facility at University of North Carolina at Chapel Hill, and (C) leptin levels by serum ELISA (ThermoFisher). One way ANOVA corrected for multiple comparisons. No significant differences were observed between the groups. n = 5–7 mice/group. (TIF) [file pone.0286470.s001.TIF]

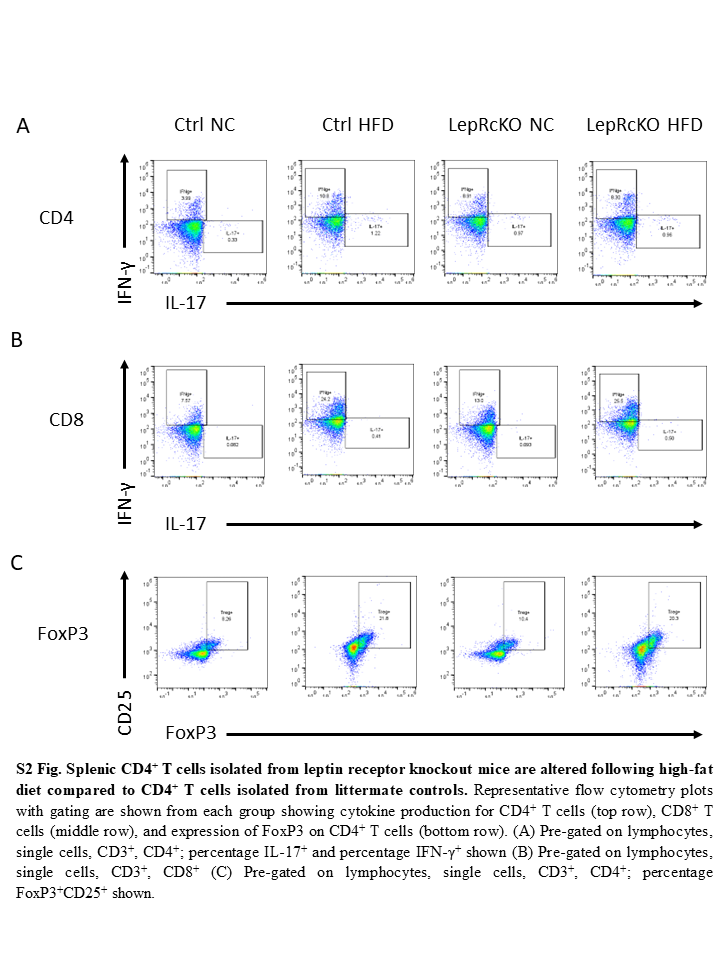

Supplement: S2 Fig — Representative flow cytometry plots with gating are shown from each group showing cytokine production for CD4+ T cells (top row), CD8+ T cells (middle row), and expression of FoxP3 on CD4+ T cells (bottom row). (A) Pre-gated on lymphocytes, single cells, CD3+, CD4+; percentage IL-17+ and percentage IFN-γ+ shown (B) Pre-gated on lymphocytes, single cells, CD3+, CD8+ (C) Pre-gated on lymphocytes, single cells, CD3+, CD4+; percentage FoxP3+CD25+ shown. (TIF) [file pone.0286470.s002.TIF]

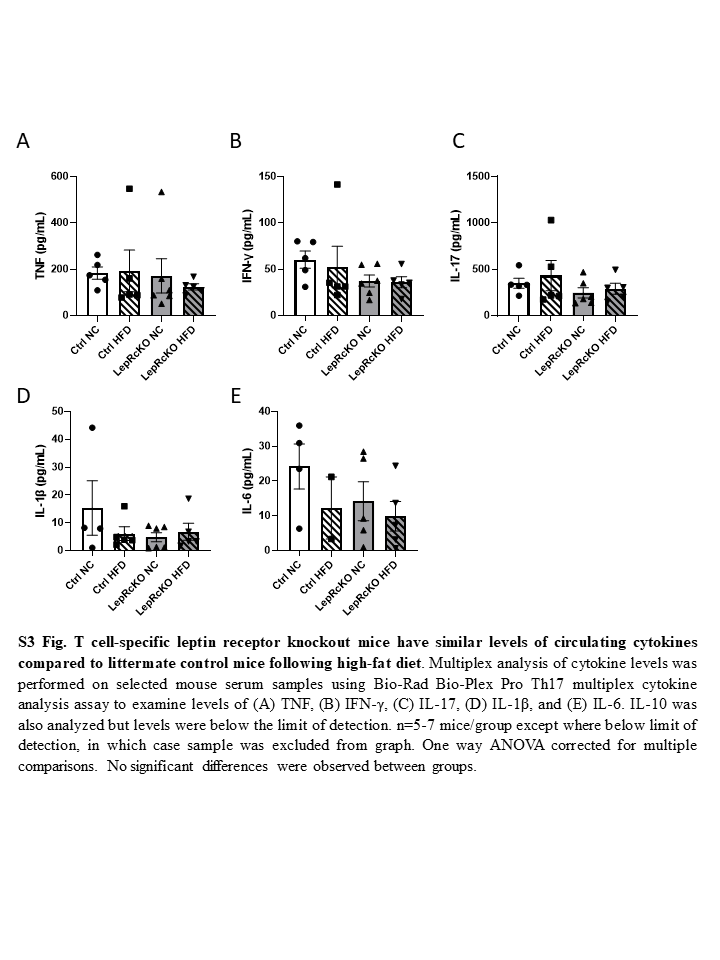

Supplement: S3 Fig — Multiplex analysis of cytokine levels was performed on selected mouse serum samples using Bio-Rad Bio-Plex Pro Th17 multiplex cytokine analysis assay to examine levels of (A) TNF, (B) IFN-γ, (C) IL-17, (D) IL-1β, and (E) IL-6. IL-10 was also analyzed but levels were below the limit of detection. n = 5–7 mice/group except where below limit of detection, in which case sample was excluded from graph. One way ANOVA corrected for multiple comparisons. No significant differences were observed between groups. (TIF) [file pone.0286470.s003.TIF]
