# Supplementary material for: Effects of T cell leptin signaling on systemic glucose tolerance and T cell responses in obesity
Source: PLoS One. 2023 Jun 5;18(6):e0286470. doi: 10.1371/journal.pone.0286470 (PMC10241364; doi:10.1371/journal.pone.0286470)
Supplement: Attachment — Submitted filename: Response to reviewers.docx [file pone.0286470.s005.docx]

**Response to reviewers**

We thank the reviewers for their careful review of our manuscript. We have addressed all reviewer comments below and now present an improved manuscript for review.

Reviewer #1:

1. *Authors need to revise the structure of results section. Please structure with proper subsections and describe your results separately; not only with Figures legends/captions. Also, figure legends should be submitted in a different section or along with figures.*

Thank you for this suggestion. We agree that adding subsections would improve the structure of the manuscript. In response to this comment, subsections were added to the results section. Figure legends are placed in the text as per PLOS manuscript guidelines.

2. *Why you didn't check lipid profile? What about systemic effects of hypercholesterolemia? It greatly trigger cardiovascular and immune response. Please justify.*

Thank you for this comment. We agree that systemic effects of hypercholesterolemia could be impacted in our mouse model and subsequently influence T cell responses. In response to this comment, we performed lipid analyses on serum samples from this study. Please see Supplemental Figure 2. We did not see any significant differences in total cholesterol or triglyceride levels between the experimental groups.

3. *You could also take pictures of spleen from each mice. Could help justify splenomegaly!*

While we did not have photographs of the spleens from these mice, we did record spleen weights and weight of visceral adipose tissue, so we have included that information in the graphs below.

4. *Study could elaborate several other factors and parameters of systemic inflammation. Why didn't you do that. Please justify other possible limitations.*

Thank you for this comment. We agree that more markers and parameters of systemic inflammation would be informative. In response to this comment, we performed multiplex analysis on serum samples from our mouse cohort. We assessed serum levels of TNF, IFN-γ, IL-17, IL-1β, and IL-6. We did not find any significant differences in serum cytokine levels between the groups. We also performed a leptin ELISA to measure serum leptin levels in these mice. This data is included in Supplemental Figures 1 and 3.

Reviewer #2:

1. *In this article, the authors have focused on understanding the leptin signaling pathway driving T-cell inflammation in obesity. This is a well-written manuscript with minor correction that needs to be addressed,*

*Line 61 Expand Rag-/-
Quote reference for all the protocols followed in the method section
Line 148 quote the company
Include Scatter plot for flow cytometry
Give a line of reason for every result stated in the study. For example, why are the metabolism and function affected in CD4+ T cells when compared to CD8+ T cells?
Check for grammatical mistakes throughout the manuscript*

We thank the reviewer for these helpful comments. In response to the comments from this reviewer, changes to the text were made, including expanding on “Rag-/-” to clarify, referencing our publications where methods have been published previously, and including several sentences explaining the results presented. We also included flow cytometry scatter plots in Supplemental Figure 2.
